# Implant survival of 662 dual-mobility cups and 727 constrained liners in primary THA: small femoral head size increases the cumulative incidence of revision

**DOI:** 10.1080/17453674.2021.1939597

**Published:** 2021-07-09

**Authors:** Oskari Pakarinen, Olli Lainiala, Aleksi Reito, Perttu Neuvonen, Keijo Mäkelä, Antti Eskelinen

**Affiliations:** a Coxa Hospital for Joint Replacement, and Faculty of Medicine and Health Technology, University of Tampere, Tampere; b Department of Radiology, Tampere University Hospital, Tampere; c Department of Orthopedics and Traumatology, Turku University Hospital, and University of Turku, Turku, Finland

## Abstract

Background and purpose — In total hip arthroplasty (THA), the risk for dislocation can be reduced using either dual-mobility cups (DMCs) or constrained liners (CLs). There are few studies comparing these concepts in primary THA. Therefore, we compared the cumulative incidence of revision in primary THA patients treated with DMC or CL with varying head sizes with conventional THA patients as reference group.

Patients and methods — We performed a cohort study based on the Finnish arthroplasty register, comparing DMCs and CLs operated over the period 2000–2017. DMCs were divided into 2 groups based on the implant design: “DMC Trident” group (n = 399) and “DMC Others” group (n = 263). CLs were divided based on the femoral head size: “CL 36 mm” group (n = 425) and “CL < 36 mm” group (n = 302). All conventional primary THAs operated on in 2000–2017 with 28–36 mm femoral head were included as control group (“Conventional THA” group, n = 102,276). Implant survival was calculated by the corresponding cumulative incidence function with revision as the endpoint and death as competing event. Also, the prevalence of different reasons for revision was compared.

Results — The 6-year cumulative incidence function estimates for the first revision were 6.9% (95% CI 4.0–9.7) for DMC Trident, 5.0% (CI 1.5–8.5) for DMC Others, 13% (CI 9.3–17) for CL < 36 mm, 6.3% (3.7–8.9) for CL 36 mm, and 4.7% (CI 4.5–4.8) for control group (conventional THA). The prevalence of dislocation revision was high (5.0%, CI 2.9–8.2) in the CL < 36 mm group compared with other groups.

Interpretation — The DMC and CL 36 mm groups had promising mid-term survival rates, comparable to those of primary conventional THA group. The revision rate of CLs with < 36 mm head was high, mostly due to high prevalence of dislocation revisions. Therefore, CLs with 36 mm femoral head should be preferred over smaller ones.

Dislocation is the most common complication after primary total hip arthroplasty (THA). Moreover, according to data from large national registers, dislocation is the most common reason for revision during the first postoperative year (Australian Orthopaedic Association 2019, National Joint Registry 2019). In primary THA, the prevalence of dislocation varies from 0.4% to 4.1% (Blom et al. 2008, Itokawa et al. 2013, Ravi et al. 2014, Klasan et al. 2019, Pakarinen et al. 2020, Hermansen et al. 2021). Recently, the role of implants that increase hip stability has been emphasized for patients who are at high risk of dislocation (Hernigou et al. 2010, 2016, Nessler et al. 2020). These implants use either larger femoral heads or have been specifically designed to prevent dislocations, as in dual-mobility cups (DMC) and constrained acetabular liners (CL) (Guyen 2017, Van der Merwe 2018, Reina et al. 2019). The use of DMCs gained worldwide popularity during the 2010s (American Joint Replacement Registry 2018, Bloemheuvel et al. 2019).

The advantage of DMCs with regard to hip stability is that larger femoral heads can be used. Despite the small femoral head in the inner bearing, a large mobile polyethylene liner can be used as an articulating head for the outer bearing (Terrier et al. 2017). Although CLs have differences in design, enhanced stability is achieved by more than a hemispheric coverage of the liner and a metallic reinforcement ring, which mechanically secures the head into the liner. The disadvantage of CLs is that the range of motion (ROM) of the hip joint is limited by their structure. This can lead to impingement, breakage of the locking mechanism, and increased wear (Burroughs et al. 2001). Therefore, CLs have traditionally been reserved for a very limited group of patients, especially those with abductor insufficiency (Herman et al. 2019).

Even though the results of DMCs and CLs in both primary and revision THA have previously been studied separately, there is a scarcity of literature in which these concepts are compared with each other in primary THA. Further, although the effect of femoral head size on hip stability has been widely studied, this is not the case with CLs. In this study, we compared the cumulative incidence of revision in primary THA patients treated with DMC or CL with varying head sizes with conventional THA patients as reference group. 

## Patients and methods

### Data sources

This study is based on data from the Finnish Arthroplasty Register (FAR), which has 95% completeness of all primary THAs and 81% completeness of all revision THAs performed in Finland (Finnish Arthroplasty Register 2020). All orthopedic units are obligated to provide essential information to the Finnish National Institute of Health and Welfare. Death dates were obtained from the Population Information System maintained by the Finnish Population Register Centre.

### Study population

**Figure 1.**
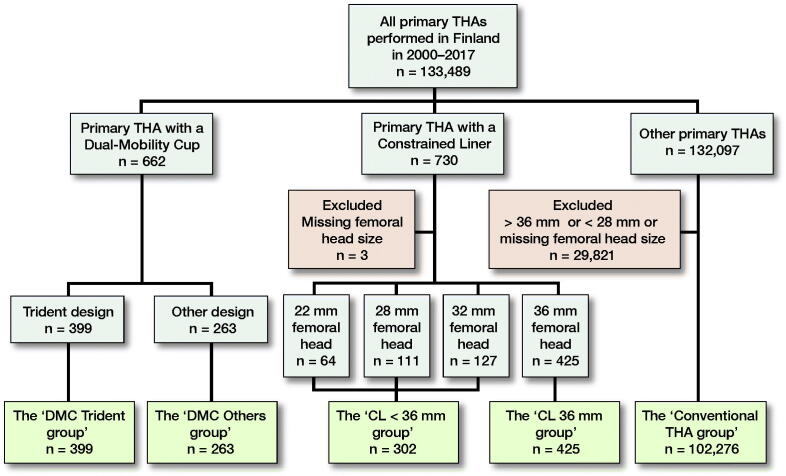
Flow diagram of the study. THA = total hip arthroplasty, DMC = dual-mobility cup, CL = constrained liner.

We identified all primary THAs performed in Finland between January 1, 2000 and December 31, 2017 in which either a DMC or CL cup was used. The final data included 662 hips with DMCs and 727 hips with uncemented cups with CLs, which represents 1.0% of the primary THA patients during that time period (Figure 1). We included all cemented and uncemented DMCs with either 22 mm or 28 mm metal or ceramic inner femoral heads and larger outer dual-mobility liners of all sizes. DMCs were split into 2 groups, because the most common DMC in our data, i.e., the Trident (Stryker, Mahwah, NJ, USA), differs markedly from the other designs. Unlike the other designs that consist of a 1-piece metal cup articulating against a large dual-mobility head, the Trident is a classical modular uncemented cup, into which a metal liner is inserted. As a result, size of the dual-mobility head is smaller in Trident than in the 1-piece cup DMC designs. The Trident has also been associated with a risk of mal-insertion of the liner (Langdown et al. 2007, Romero et al. 2020). The “DMC Trident group” included all Trident DMCs (n = 399), and the “DMC Others group” (n = 263) included the rest of the DMCs. To assess the role of head size in the survivorship of CLs, 2 different groups were formed: the “CL < 36 mm group” (n = 302), which included 22 mm (n = 64), 28 mm (n = 111), and 32 mm (n = 127) femoral heads, and the “CL 36 mm group” that comprised 36 mm femoral heads only (n = 425). In the data, there were no CLs with femoral heads larger than 36 mm. The most commonly used DMCs and CLs are listed in Table 1. The patients were operated on in 2012–2017 in the DMC Trident group (median follow-up 2.4 years), 2007–2017 in the DMC Others group (3.3 years), 2000–2017 in the CL < 36 mm group (2.3 years), and 2005–2017 in the CL 36 mm group (2.4 years). We included all conventional primary THAs performed in 2000–2017 with a 28–36 mm sized femoral head as a control group (n = 102,276, median follow-up 5.5 years). Operations with < 28 mm were excluded from the control group because the smallest head sizes have been associated with high risk of dislocation, and > 36 mm heads were excluded because we assume that they mostly consist of large metal-on-metal heads that are known to have a high revision rate because of the adverse reaction to metal debris (Berry et al. 2005, Lainiala et al. 2019).

**Table 1.** Cup and liner designs included in the study cohort

	Dual-mobility (Trident)	Dual-mobility (Others)	CL < 36 mm	CL 36 mm	Conventional THA
Cup design (n)	Trident (399)	Restoration ADM (152)	Continuum (98)	Vision Ringloc (293)	Pinnacle (17,136)
*(Liner design %)*	*(MDM 92%)*	*(Restoration ADM 100%)*	*(Continuum 100%)*	*(Freedom 100%)*	Trident (12,553)
	*(Restoration 8%)*	Novae E (80)	Trident (80)	Regenerex (72)	Contemporary (12,314)
		*(Novae E 100%)*	*(Trident 90%)*	*(Freedom 100%)*	Continuum (11,895)
		Avantage (15)	*(Data missing 10%)*	Continuum (15)	R3 (7,515)
		*(Avantage 100%)*	Pinnacle (44)	*(Continuum 100%)*	Reflection (6,120)
		Others (16)	*(Pinnacle 100%)*	G7 (12)	Vision Ringloc (4,941)
			Trabecular metal (35)	*(Freedom 100%)*	ABG (3,885)
			*(Trabecular metal 72%)*	Pinnacle (11)	Exeter (2,571)
			*(Vision Ringloc 17%)*	*(Pinnacle 100%)*	Exceed (2,047)
			*(Continuum 9%)*	Others (22)	Lubinus (1,572)
			Vision Ringloc (23)		G7 (1,183)
			*(Vision Ringloc 100%)*		Others (18,544)
			Others (15)		
Total	399	263	302	425	102,276

DMC = dual-mobility cup, CL = constrained liner. Cup and liner designs listed with manufacturer: Trident (Stryker, Mahwah, NJ, USA); Restoration (Stryker); Novae E (Fischer Medical, Glostrup, Denmark); Avantage (Zimmer Biomet, Warsaw, IN, USA); Continuum (Zimmer Biomet); Pinnacle (Depuy Orthopaedics, Warsaw, IN, USA); Trabecular Metal (Zimmer Biomet); Vision RingLoc (Zimmer Biomet); Regenerex (Zimmer Biomet); G7 (Zimmer Biomet); Freedom (Zimmer Biomet); Contemporary (Stryker); R3 (Smith & Nephew, Memphis, TN, USA); Reflection (Smith & Nephew, London, UK); ABG (Stryker); Exeter (Stryker); Exceed (Zimmer Biomet); Lubinus (Link, Hamburg, Germany).

### Statistics

Follow-up started on the day of primary THA and ended on the day of revision, death, or June 10, 2018 (the date of data collection), whichever came first. Revision was defined as a new surgical procedure, including partial or complete removal or exchange of any implant component. The indications for revision specified in the FAR database were dislocation, periprosthetic femoral fracture (PFF), aseptic loosening, deep infection, pain, and other reasons.

The overall mortality was high in all study groups compared with the endpoint of interest, i.e., the number of revisions (Table 2). Our original plan was to analyze the risk for revision using the Fine–Gray competing risk regression with first revision of the primary THA as the primary endpoint and death as the competing endpoint. Age, sex, primary diagnosis, femoral and acetabular fixation were supposed to be included in the analysis. However, multiple proportional hazards assumption violations were found after the inspection of the corresponding log-survival against log-time across categorized covariate levels, which would have made the interpretation of the results difficult. Moreover, it was evident that even after the adjustments there would be a substantial amount of unmeasured confounding as a result of selection bias. Therefore, we decided to calculate only the implant survival rate using corresponding cumulative incidence function (CIF) with patient death as a competing event and accept that the patient-related factors could not be reliably adjusted. The first sensitivity analysis was similar to the main one, but the endpoint was revision for any reason except PFF because the increased risk for this type of complication is mostly associated with the type of femoral stem and not the acetabular component (Thien et al. 2014). In the second sensitivity analysis, the different head sizes within the CL < 36 mm group were compared with each other.

**Table 2.** Patient demographics. Values are count (%) unless otherwise specified

	DMC	DMC	CL	CL	Conventional
	(Trident)	(Others)	< 36 mm	36 mm	THA
	n = 399	n = 263	n = 302	n = 425	n = 102,276
Follow-up, years,	2.4	3.3	2.3	2.4	5.5
median (IQR)	(1.1–3.9)	(1.1–6.1)	(0.9–4.0)	(0.8–4.8)	(2.6–10)
Age, mean (SD)	71 (11)	69 (11)	73 (12)	71 (12)	67 (11)
BMI, mean (SD)	27 (5.2)	26 (5.2)	26 (5.0)	27 (5.5)	28 (4.8)
Sex					
Male	156 (39)	95 (36)	105 (35)	199 (47)	41,053 (40)
Female	243 (61)	167 (64)	197 (65)	225 (53)	61,201 (60)
Data missing	0 (0.0)	1 (0.4)	0 (0.0)	1 (0.2)	22 (0.0)
Diagnosis					
Osteoarthritis	248 (62)	147 (56)	99 (33)	109 (26)	87,199 (85)
Hip fracture	97 (24)	85 (32)	88 (29)	101 (24)	4,220 (4.1)
Other	54 (14)	31 (12)	115 (38)	215 (51)	10,857 (11)
ASA score **^a^**					
1	8 (2.8)	1 (0.8)	3 (1.8)	5 (2.7)	3,836 (12)
2	91 (32)	37 (30)	30 (18)	33 (18)	15,179 (49)
3	175 (62)	79 (64)	110 (67)	111 (60)	11,367 (37)
4	9 (3.1)	7 (5.6)	21 (13)	36 (20)	493 (1.6)
Approach **^a^**					
Posterior	198 (70)	106 (85)	165 (98)	166 (90)	24,603 (81)
Anterolateral					
(mod. Hardinge)	86 (30)	19 (15)	4 (2.4)	19 (10)	5,892 (19)
Cup fixation					
Uncemented	399 (100)	200 (76)	259 (86)	193 (45)	68,948 (67)
Cemented	0 (0.0)	49 (19)	24 (7.9)	219 (52)	29,270 (29)
Data missing	0 (0.0)	14 (5.3)	19 (6.3)	13 (3.1)	4,058 (4.0)
Femoral fixation					
Uncemented	102 (34)	114 (43)	132 (44)	230 (54)	56,593 (55)
Cemented	294 (74)	145 (55)	149 (49)	115 (27)	41,161 (41)
Data missing	3 (0.8)	4 (1.5)	21 (7.0)	80 (19)	4,522 (4.4)
Femoral head material					
Metal	323 (81)	181 (69)	270 (89)	408 (96)	68,739 (67)
Ceramic	23 (5.8)	65 (25)	15 (5.0)	6 (1.4)	33,537 (33)
Data missing	53 (13)	17 (6.5)	17 (5.6)	11 (2.6)	0 (0.0)
Total mortality	34 (8.5)	38 (14)	99 (33)	212 (50)	22,180 (22)
Revision surgery	23 (5.8)	9 (3.4)	39 (13)	23 (5.4)	6,069 (5.9)
Reason for revision					
Aseptic loosening	2 (0.5)	1 (0.4)	2 (0.7)	3 (0.7)	1,307 (1.3)
Deep infection	7 (1.8)	2 (0.8)	6 (2.0)	10 (2.4)	728 (0.7)
PFF	7 (1.8)	3 (1.1)	11 (3.6)	4 (0.9)	769 (0.8)
Dislocation	1 (0.3)	0 (0)	15 (5.0)	1 (0.2)	1,422 (1.4)
Pain only	0 (0)	1 (0.4)	0 (0)	0 (0)	71 (0.0)
Others	5 (1.3)	2 (0.8)	3 (1.0)	1 (0.2)	895 (0.9)
Unknown reason	1 (0.3)	0 (0)	2 (0.7)	4 (0.9)	877 (0.9)

DMC = dual-mobility cup, CL = constrained liner, IQR = interquartile range, BMI = body mass index, ASA score = American Society of Anesthesiologists score, PFF = periprosthetic femoral fracture.

a Data available only for patients operated on after 2014. BMI coverage in data: 66% in DMC Trident group, 47% in DMC Others group, 47% in CL < 36 mm group, 33% in CL 36 mm group, and 28% in Conventional THA group.A

We calculated 95% confidence intervals (CI) for CIF graphs. CI for proportions were calculated using Wilson score interval. The analyses were performed using IBM SPSS 25.0 (IBM Corp, Armonk, NY, USA) and R statistical software (R Centre for Statistical Computing, Vienna, Austria).

### Ethics, funding, and potential conflicts of interest

In accordance with Finnish regulations, informed patient consent was not required as the patients were not contacted. This work was supported by the competitive research funds of Pirkanmaa Hospital District, Tampere, Finland (representing governmental funding), Orion Research Foundation, Vappu Uuspää Foundation, and Finnish Research Foundation for Orthopaedics and Traumatology. The sources of funding had no role at any stage of the study. Individual potential conflict of interests: OP, OL, AR, PN, KM: None. AE: Zimmer Biomet, paid lectures; Depuy Synthes and Zimmer Biomet, institutional research support (not related to current study).

## Results

Patient demographics of the 4 study groups and the control group of conventional THAs are summarized in Table 2. The specific primary reason for operation has been recorded in FAR data since 2014. Therefore, the more detailed indications for primary THA for patients operated on in 2014–2017 are presented in Table 3 (see Supplementary data).

**Figure 2.**
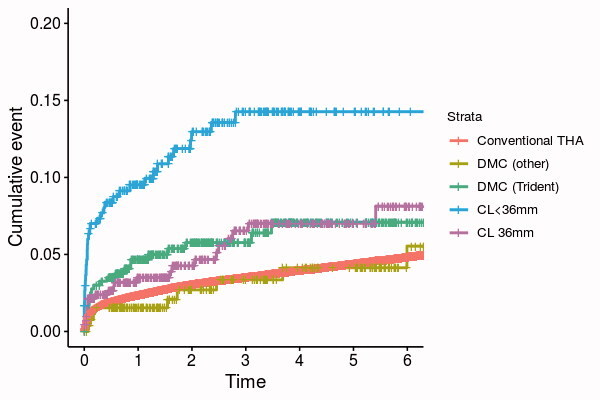
Cumulative incidence of revision.

In the 4 study groups, 94 hips were revised during the follow-up (6.8%, CI 5.6–8.3). The most common reasons for revision were PFF (n = 25, 1.8%), deep infection (n = 25, 1.8%), and dislocation (n = 17, 1.2%). In the Conventional THA group, the overall revision rate was 5.9% (n = 6,069, CI 5.8–6.1), and the leading causes of revision were dislocation (n = 1,422, 1.4%) and aseptic loosening (n = 1,307, 1.3%). The 1-year postoperative mortality was 3.5% in the DMC Trident group, 5.7% in the DMC Others group, 11% in the CL < 36 mm group, 20% in the CL 36 mm group, and 1.8% in the Conventional THA group. The cumulative incidence of the first revision of the study groups is presented in Figure 2.

At 6 years postoperatively, the CIF estimate of the first revision was 6.9% (CI 4.0–9.7) for DMC Trident, 5.0% (CI 1.5–8.5) for DMC Others, 13% (CI 9.3–17) for CL < 36 mm, 6.3% (CI 3.7–8.9) for CL 36 mm, and 4.7% (CI 4.5–4.8) for Conventional THA (Table 4). During the same 6-year period, the CIF estimate of death was 13% (CI 8.3–18) for DMC Trident, 17% (CI 11–23) for DMC Others, 37% (CI 29–44) for CL < 36mm, 54% (CI 48–60) for CL 36 mm, and 12% (CI 12–12) for Conventional THA. The CIF estimates are visually presented in Figure 3 (see Supplementary data).

**Table 4.** Cumulative incidence function estimates at 1, 3, and 6 years for the first revision and death with 95% confidence intervals

	DMC	DMC	CL	CL	Conventional
	(Trident)	(Others)	< 36 mm	36 mm	THA
**All-cause revision**					
Risk of revision					
1 year	4.6 (2.5–6.7)	1.5 (0.4–3.0)	9.3 (6.0–13)	3.3 (1.6–5.0)	2.3 (2.3–2.4)
3 years	5.7 (3.3–8.0)	3.2 (0.8–5.5)	13 (9.3–17)	5.5 (3.2–7.7)	3.5 (3.4–3.6)
6 years	6.9 (4.0–9.7)	5.0 (1.5–8.5)	13 (9.3–17)	6.3 (3.7–8.9)	4.7 (4.5–4.8)
Risk of death					
1 year	3.2 (1.4–5.0)	5.7 (2.8–8.6)	8.2 (5.0–11)	18 (14–22)	1.3 (1.2–1.4)
3 years	9.0 (5.7–12)	10 (6.3–14)	20 (15–25)	34 (29–39)	4.6 (4.4–4.7)
6 years	13 (8.3–18)	17 (11–23)	37 (29–44)	54 (48–60)	12 (12–12)
**All-cause revision (PFF excluded)**					
Risk of revision					
1 year	2.8 (1.2–4.5)	1.1 (0.0–2.4)	6.7 (3.9–9.6)	2.9 (1.3–4.4)	2.0 (1.9–2.0)
3 years	3.9 (1.9–5.9)	2.8 (0.5–5.0)	9.5 (6.0–13)	4.4 (2.3–6.5)	3.0 (2.9–3.1)
6 years	5.1 (2.5–7.7)	3.2 (0.8–5.5)	9.5 (5.9–13)	5.3 (2.9–7.7)	4.1 (4.0–4.2)
Risk of death					
1 year	3.5 (1.6–5.4)	6.1 (3.1–9.1)	9.4 (5.9–13)	18 (15–22)	1.4 (1.3–1.5)
3 years	9.4 (6.0–13)	11 (6.6–15)	22 (16–27)	35 (30–40)	4.7 (4.6–4.8)
6 years	13 (8.6–18)	17 (12–23)	39 (31–47)	55 (49–61)	12 (12–12)

When revisions for PFF were excluded, the 6-year CIF estimate of the first revision was 5.1% (CI 2.5–7.7) for DMC Trident, 3.2% (CI 0.8–5.5) for DMC Others, 9.5% (CI 5.9–13) for CL < 36 mm, 5.3% (CI 2.9–7.7) for CL 36 mm, and 4.1% (CI 4.0–4.2) for Conventional THA (Table 4). The reasons for revision surgery are presented in Table 2. Dislocation was the most common reason for revision in the CL < 36 mm group (5.0% prevalence, CI 2.9–8.2), but a very rare reason for revision in the CL 36 mm (0.2%, CI 0.01–1.5), DMC Trident (0.3%, CI 0.01–1.6), and DMC Others (0.0%, CI 0.0–1.8) groups. In the Conventional THA group, the prevalence of dislocation revision was 1.4% (CI 1.3–1.5) (Table 2).

When the 22 mm, 28 mm, and 32 mm head sizes within the CL < 36 mm group were compared, the cumulative incidence estimates of the first revision were similar regardless of femoral head size (Table 5, see Supplementary data). 

## Discussion

We found that the cumulative incidence of revision in 6-year follow-up was comparable with the conventional THA patients in the DMC Others group, and only slightly higher in the DMC Trident and CL 36 mm groups. However, in the CL < 36 mm group the revision rate was remarkably higher. Dislocation was a major cause of revision in CL < 36 mm group, but a rarity in other study groups. In total, DMCs and CLs represented 1.0% of the primary THAs implanted in Finland during the study period, indicating they were used in a selected population, presumably with high risk of dislocation.

The survivorship of DMCs has been reported to be comparable to that of conventional THA in primary THA with mid-term follow-up (Kreipke et al. 2019), whereas DMCs have been associated with low dislocation and revision rates in primary THA with dislocation-prone patients (Harwin et al. 2017, Jones et al. 2019). Some implant-related complications, such as intraprosthetic dislocations, have, however, been reported (Addona et al. 2019). In our study, the mid-term survival rate of the DMC Others group was comparable to the conventional THA patients. No dislocation revisions occurred even though a third of the patients in the former group were operated on for hip fracture. In the DMC Trident group the revision estimates were only slightly higher, and only 1 hip (0.3%) was revised for dislocation. Due to the register-based study setting, we were unable to verify the reasons why surgeons chose either DMCs or CLs in primary THAs. We can only assume that the reason was an anticipated high risk for dislocation in most of the cases. Nonetheless, the indications may have differed because both the mortality and the proportion of patients operated on for reasons other than osteoarthritis or hip fracture differ between the DMC and CL groups. Since 2014, patients operated on for tumor comprised a third of the patients in the CL 36 mm group (see Supplementary data 1), which partially explains the high mortality rate in this group. Regardless of the proportion of high-risk patients in our data, our results are in line with other recent studies and do not oppose the idea of using DMCs in primary THA for patients who have a higher risk for dislocation. Still, longer follow-up is needed to see how well these implants actually bear the test of time.

The biggest advantage with larger femoral heads is the decreased risk of dislocation (Berry et al. 2005, Hailer et al. 2012, Howie et al. 2012, Kostensalo et al. 2013). Thoms and Marwin (2008) have suggested that femoral head size ought to be maximized when a constrained liner is used. The rationale for this is to increase the head-to-neck ratio and lever-out distance and thus decrease the risk for impingement and dislocation (Soong et al. 2004, Brown et al. 2014). However, no prior studies have reported whether increasing the head size with CLs actually results in better outcome.

Recent studies have reported good survival rates for CLs in primary THA (Clave et al. 2016, Gill et al. 2016, Karvonen et al. 2020). In our study, the overall 6-year survival rate of CLs with 36 mm head was also promising, but there were considerably more revisions when CLs were used with < 36 mm head. This difference is mostly explained by higher rates of revisions for dislocation and PFF in the CL < 36 mm group. These results may indicate that a large enough femoral head with CLs allows a wide enough ROM that prevents impingement and is therefore not as prone to dislocations. The revision estimates in the CL 36 mm group were only marginally higher compared with conventional THA patients, even though the patients in the former group were remarkably more morbid on average. Still, a failed THA with CL may predispose to recurrent revision surgeries (Hellman et al. 2018). Thus, more studies are needed before the use of CLs can be recommended to prevent dislocations in primary THA for patients who do not have an obvious, strong predisposing factor for dislocation, such as abductor muscle deficiency, tumor resection, or femoral neck fracture. In patients with an increased risk of dislocation but without abductor deficiency, DMC might be a safer option as it provides better impingement-free ROM, and thus has smaller risk for mechanical failure.

In a recent study, the survivorship of the Freedom constrained acetabular liner (Zimmer Biomet) was similarly compared with conventional primary THA designs (Karvonen et al. 2020). In our study, 89% of the CLs with 36 mm heads were Freedom as it is the most commonly used CL design in Finland. Freedom liners enable the use of a 36 mm head in cup sizes as small as 50 mm, whereas the next smallest cup accepting a 36 mm head in our data is the Pinnacle 56 mm (Karvonen et al. 2020). Because the survival rate in the CL 36 mm group was excellent irrespective of the CL cup design, it seems that the larger head size, not the cup design itself, may to be the key to success when CLs are used in primary THA. However, for some patients even the use of 50 mm diameter cup is impossible and therefore the use of CL with 36 mm head is not an option.

We acknowledge a few weaknesses in this study. Because not all DMCs and CLs have identical designs, there could be implant-related factors that have affected the risk for revision that we are not aware of. The rather short mean follow-up limits the interpretation of our long-term survivorship comparison. Because of the heterogeneity in the study population, we reported only unadjusted CIF estimates. Even after the available factors had been adjusted, the comparison would not have been equal because the indications for the use of CL or DMC in primary THA are different compared with conventional THA implants. There were differences in mortality, distribution of ASA score, and the primary reason for operation between the DMCs and CLs, implying that there is confounding by indication also between these groups (see Table 2). Because the mortality and the number of patients operated on for reasons other than osteoarthritis were highest in the CL 36 mm group, it is unlikely that the comorbidities would explain the inferior results in the CL < 36 mm group compared with the CL 36 mm group. Because of the limitations in the data, the impact of unmeasured confounding must be considered in the interpretation of the results. As this was a register study, we could not comprehensively assess the clinical outcome of the operations (e.g., patient-reported outcome measures).

## Conclusion

The DMC Other group showed a comparable revision rate with conventional THA implants in 6-year follow-up, and the revision rate for the DMC Trident and CL 36 mm groups was only slightly higher. Conversely, the 6-year revision rate was clearly higher in the CL < 36 mm group. The difference was mostly explained by dislocations because revision for dislocation was a very rare event in both DMC groups and in the CL 36 mm group, whereas it was the most common type of revision in the CL < 36 mm group. The prevalence of PFF revision was also highest in the CL < 36 mm group. The good overall survival rate and low number of dislocation revisions with DMCs support the increased use of these devices over recent years. However, studies with long-term follow-up are still needed. According to our results, it seems that enlarging the femoral head with CLs enhances the survival rate of the implant. Therefore, we recommend that when a CL is used, a 36 mm femoral head should be preferred over a smaller head to avoid complications, especially dislocations.

## Supplementary Material

Supplemental Material

## Supplementary data

Tables 3 and 5 and Figure 3 are available as supplementary data in the online version of this article, http://dx.doi.org/10.1080/17453674.2021.1939597
