# Supplementary material for: Implant survival of 662 dual-mobility cups and 727 constrained liners in primary THA: small femoral head size increases the cumulative incidence of revision
Source: Acta Orthop. 2021 Jul 9;92(6):658–64. doi: 10.1080/17453674.2021.1939597 (PMC8641668; doi:10.1080/17453674.2021.1939597)
Supplement: Supplemental Material [file IORT_A_1939597_SM9101.pdf]

# Supplementary data

**Table 3. More specific indications for primary THA (includes only patients operated in years 2014–2017). Values are count (%)**

| Diagnosis | DMC (Trident) n = 306 | DMC (Others) n = 134 | CL < 36 mm n = 185 | CL 36 mm n = 203 | Conventional THA n = 34,557 |
|---|---|---|---|---|---|
| Osteoarthritis | 188 (61) | 50 (37) | 62 (34) | 53 (26) | 29,218 (85) |
| Hip fracture | 84 (27) | 68 (51) | 60 (32) | 34 (17) | 1,748 (5.1) |
| Avascular caput necrosis | 17 (5.6) | 6 (4.5) | 8 (4.3) | 4 (2.0) | 953 (2.8) |
| Developmental dysplasia of the hip | 2 (0.7) | – | 3 (1.6) | 3 (2.2) | 503 (1.5) |
| Failed osteosynthesis after hip fracture | 2 (0.7) | 2 (1.5) | 23 (12) | 14 (6.9) | 53 (0.2) |
| Secondary osteoarthritis after hip fracture | – | 1 (0.7) | 1 (0.5) | 5 (2.5) | 100 (0.3) |
| Inflammatory joint disease | 3 (1.0) | 3 (2.2) | – | – | 550 (1.6) |
| Purulent arthritis | – | – | – | 1 (0.5) | 12 (0.03) |
| Childhood joint disease | – | – | – | – | 108 (0.3) |
| Tumor | – | 1 (0.7) | 4 (2.2) | 65 (32) | 41 (0.1) |
| Other disease | 1 (0.3) | – | 10 (5.4) | 8 (3.9) | 169 (0.5) |
| Data missing | 9 (2.9) | 3 (2.2) | 14 (7.6) | 16 (7.9) | 1,102 (3.2) |

**Table 5. Cumulative incidence function estimates at 1, 3, and 6 years of first revision and death with 95% confidence intervals for the CL< 36 mm group stratified by the femoral head size**

| | CL 22 mm n = 64 | CL 28 mm n = 111 | CL 32 mm n = 127 |
|---|---|---|---|
| Risk of revision | | | |
| 1 year | 11 (3.2–19) | 10 (4.3–16) | 8.0 (3.2–13) |
| 3 years | 13 (4.4–21) | 14 (6.8–20) | 14 (7.1–21) |
| 6 years | 13 (4.4–21) | 14 (6.8–20) | 14 (7.1–21) |
| Risk of death | | | |
| 1 year | 9.4 (2.2–17) | 6.5 (1.8–11) | 9.1 (3.9–14) |
| 3 years | 27 (15–39) | 17 (9.0–26) | 17 (9.6–24) |
| 6 years | 42 (28–56) | 31 (17–45) | 37 (25–49) |

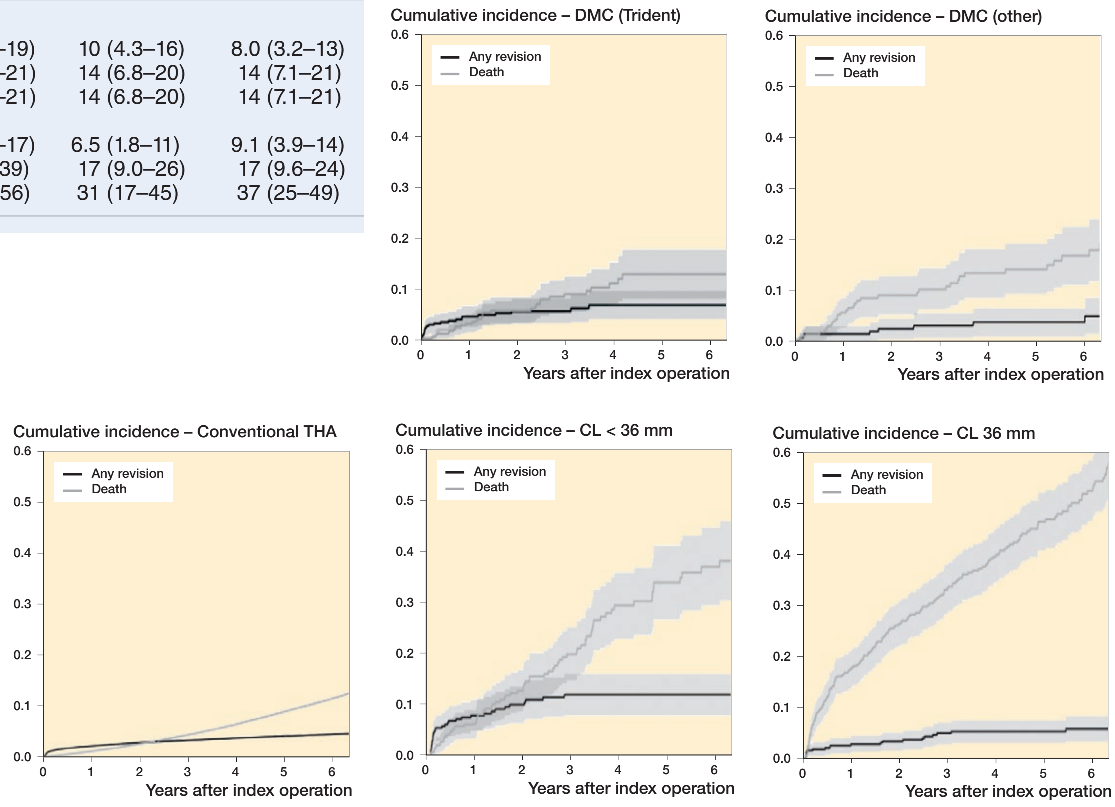


Figure 3. Cumulative incidence function estimates of the first revision with death as competing event.
